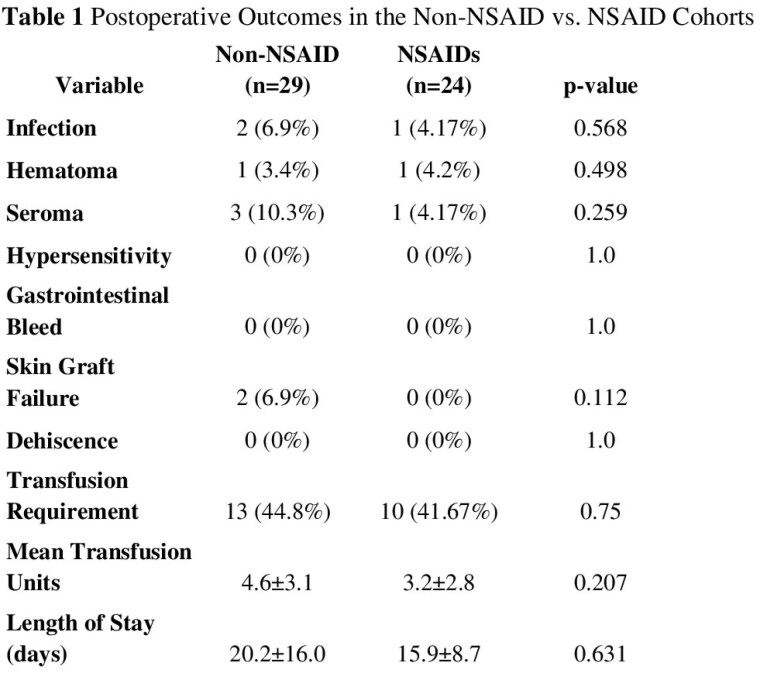# 32 Ibuprofen Is Not Associated with an Elevated Bleeding Risk in Skin Grafting After Burn Injury

**DOI:** 10.1093/jbcr/iraf019.032

**Published:** 2025-04-01

**Authors:** Artur Manasyan, Jordan Gasho, Eloise Stanton, Michael Kim, Maxwell Johnson, Haig Yenikomshian, Justin Gillenwater

**Affiliations:** University of California Keck School of Medicine; University of California Keck School of Medicine; University of Southern California; University of California Keck School of Medicine; University of California Keck School of Medicine; University of California Keck School of Medicine; Los Angeles General Medical Center

## Abstract

**Introduction:**

Ibuprofen, a non-steroidal anti-inflammatory drug (NSAID), is increasingly used alongside other medications to manage pain, reducing reliance on opioids. However, it can increase the risk of bleeding, a significant concern in burn surgery, which often involves substantial blood loss. This study aims to evaluate the safety of ibuprofen use in patients undergoing skin grafting.

**Methods:**

A retrospective chart review was conducted for patients admitted with acute burn injury from 01/01/2024 to 07/31/2024 who underwent skin grafting. Medical records were reviewed to identify those who received ibuprofen, and record data on demographics, clinical progression, and outcomes. The primary outcome variables included perioperative transfusion requirement, bleeding, skin graft failure, and other complications.

**Results:**

A total of 53 patients met inclusion criteria, 24 (45.2%) of whom received scheduled ibuprofen therapy during their hospitalization. The total body surface area affected was 12.3±9.3% for the non-ibuprofen group and 14.3±12.1% for the ibuprofen group (p=0.62). A total of 79.3% of patients in the non-ibuprofen group received meshed grafts compared to 79.2% in the ibuprofen group (p=0.734). Perioperative transfusion requirements were similar between the two cohorts, averaging 4.6±3.1 for the non-ibuprofen group and 3.2±2.8 units of packed red blood cells for the ibuprofen group (p=0.207). Skin graft failure (defined as need for re-grafting) occurred in 6.9% (n=2) of the non-ibuprofen cohort versus none (n=0) in the ibuprofen group (p=0.112). Postoperative complications did not vary significantly between the two groups for seroma (p=0.259) and infection (p=0.568). There were no cases of hypersensitivity or gastrointestinal bleeding.

**Conclusions:**

There is likely no increased risk of bleeding or skin graft failure in patients taking ibuprofen, suggesting that these medications may be safe in this context. Future research should focus on evaluating the effects of other NSAIDs and involve larger, randomized controlled trials to validate these findings further.

**Applicability of Research to Practice:**

Our findings support the potential use of ibuprofen in burn injury management without heightened concern for bleeding or graft complications.

**Funding for the Study:**

N/A